# An Interesting Case of Wernicke’s Encephalopathy in Co-Existence With Tropical Infections: A Case Report

**DOI:** 10.7759/cureus.72697

**Published:** 2024-10-30

**Authors:** Amukthamalyada Koduri, Jagadeswar Kakumani, Magesh Kumar S

**Affiliations:** 1 General Medicine, Saveetha Medical College, Chennai, IND

**Keywords:** leptospirosis, pregnant female, thiamine level, tropical infections, wernicke encephalopathy

## Abstract

Wernicke's encephalopathy (WE) is an acute neurological disorder arising from thiamine (vitamin B1) deficiency, characterized by a classic triad of ophthalmoplegia, ataxia, and altered mental status. While it is more common in males, it can occur in various contexts, including pregnancy. Leptospirosis, a zoonotic infection, can co-occur with other illnesses such as scrub typhus.

We present a case of a 27-year-old primigravida in her second trimester who developed WE, presenting with altered sensorium, fever, and neurological deficits. Initial examination revealed ophthalmoplegia and confusion, with MRI confirming typical findings of WE. Investigations indicated co-infection with leptospirosis and scrub typhus, along with abnormal liver function tests. Treatment with intravenous thiamine resulted in symptomatic improvement.

This case underscores the critical role of early diagnosis and treatment of WE, especially in pregnant patients with poor nutritional intake or hyperemesis gravidarum. Imaging studies, particularly MRI, enhance diagnostic accuracy. The patient’s condition was exacerbated by concurrent infections affecting liver function and thiamine reserves. WE, though rare in pregnancy, should be suspected in cases of nutritional deficiency and acute illness. Prompt recognition and thiamine supplementation are essential to prevent severe complications, including motor deficits and mortality.

## Introduction

Wernicke’s encephalopathy (WE) is an acute neurological disorder that arises from the biochemical disruption of the central nervous system (CNS) due to a deficiency of thiamine (vitamin B1) [1]. The seminal work of Campbell and Russell in the 1940s established thiamine deficiency as a key factor in the development of this encephalopathy, highlighting the significance of adequate nutritional status for neurological health [2]. The most important condition in females presenting with thiamine deficiency is hyperemesis gravidarum during pregnancy, which can be the major cause of the development of WE in those patients.

Epidemiologically, WE is more prevalent in males, with a male-to-female ratio of approximately 1.7:1, and it carries an estimated mortality rate of 17% if left untreated [3]. Clinically, WE is characterized by a classic triad of symptoms, which include ophthalmoplegia, ataxia, and altered mental status. However, this full triad is only observed in around 16% of cases, which underscores the variability in clinical presentation [4].

Leptospirosis, also referred to as Weil's disease, is a significant zoonotic infection that typically manifests as an acute febrile illness accompanied by myalgia. It is important to note that leptospirosis can co-occur with other infections, such as malaria, hantavirus, and scrub typhus, complicating diagnosis and management [5]. In the case at hand, we document a co-infection with scrub typhus, which adds complexity to the clinical picture.

We present the case of a 27-year-old primigravida in her second trimester, with a history of hyperemesis gravidarum and fever for two days, who developed encephalopathy. After thorough investigations, she was diagnosed with WE, along with the co-infection of leptospirosis and scrub typhus, illustrating the critical need for awareness of this condition, particularly in patients with potential nutritional deficiencies and infectious comorbidities.

## Case presentation

A 27-year-old woman with no known comorbidities and no significant medication history, who is a primigravida with 15 weeks’ gestation, currently on oral folic acid, is a resident of rural Tamil Nadu, India, was brought to the hospital by attenders with a history of altered sensorium since two days and low-grade fever since seven days, preceded by inability to walk, swaying, and blurring of vision. The patient has a history of multiple vomiting episodes since the beginning of her pregnancy, i.e., hyperemesis gravidarum with the most recent episode occurring two days ago. Due to this, the patient has decreased appetite and food intake. The patient is not a known alcoholic or smoker and has a normal sexual history with one partner. On examination, the patient was drowsy and disoriented with a Glasgow coma score (GCS) of E2V3M5(10/15). Vitals were stable, and examination of cardiovascular and respiratory systems was normal. On CNS examination, the patient had bilateral partial ptosis. There was hyporeflexia, and bilateral plantar reflex was mute. Fundus examination showed early papilledema changes in both eyes (left>right). Initial CT of the brain showed mild cerebral edema, with no other significant abnormalities.

Investigations revealed abnormal liver function tests with elevated liver enzymes, i.e., alanine aspartate was 71 U/L (normal range: 8-48 U/L) and alanine transaminase was 243 U/L (normal range: 7-55 U/L). Viral markers for HIV, HBV, and HCV were negative. Fever profile was evaluated in view of the prevalence of tropical infections in our area, which showed Lepto IgM and scrub IgM positive. The patient was started on thiamine at a dose of 500mg thrice daily, antibiotics ceftriaxone at a dose of 2 grams twice daily intravenously, and doxycycline 100mg oral twice daily before further investigations in suspicion of WE. The patient showed mild improvement with this treatment.

**Figure 1:**
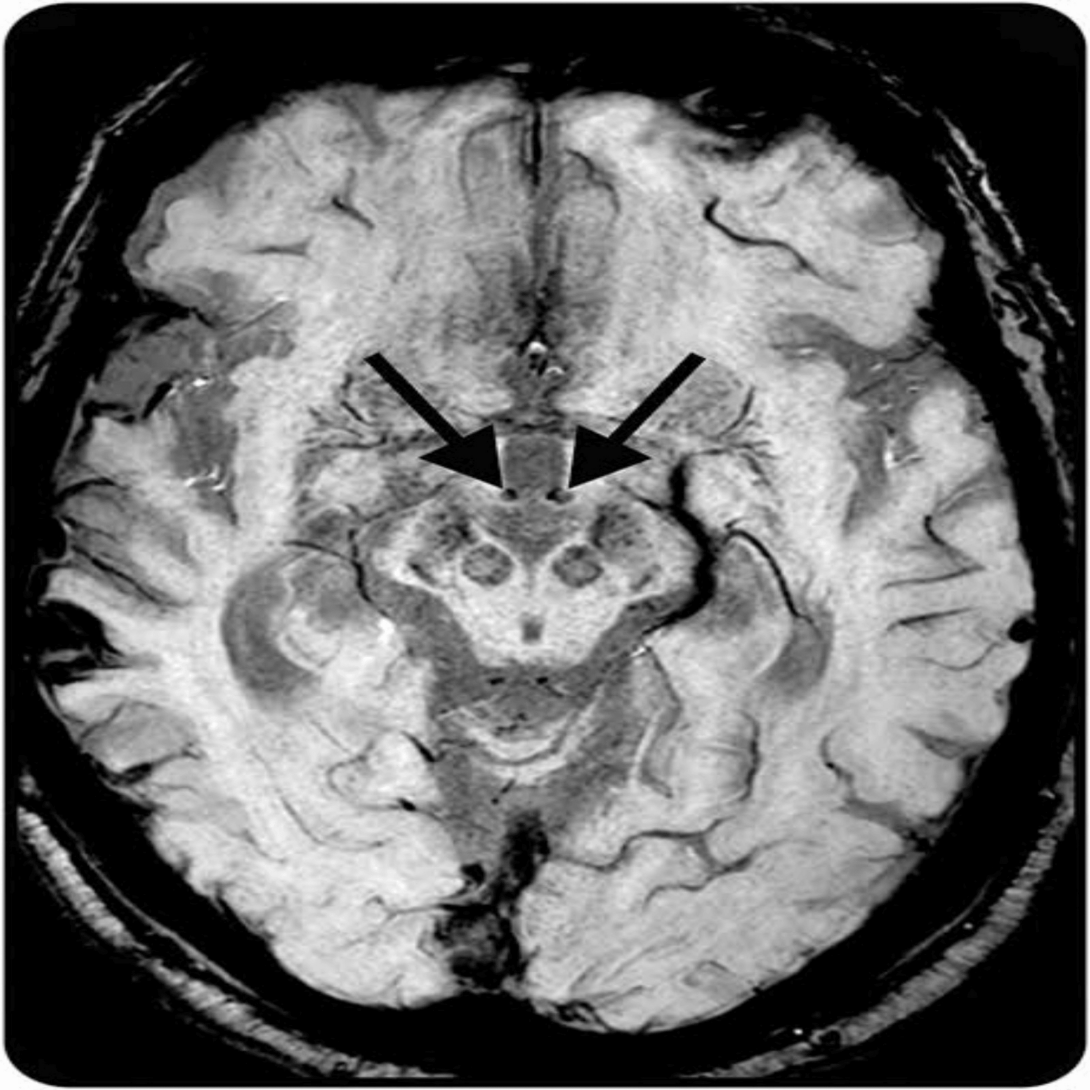
T2-weighted MRI image showing features of Wernicke’s encephalopathy

**Figure 2:**
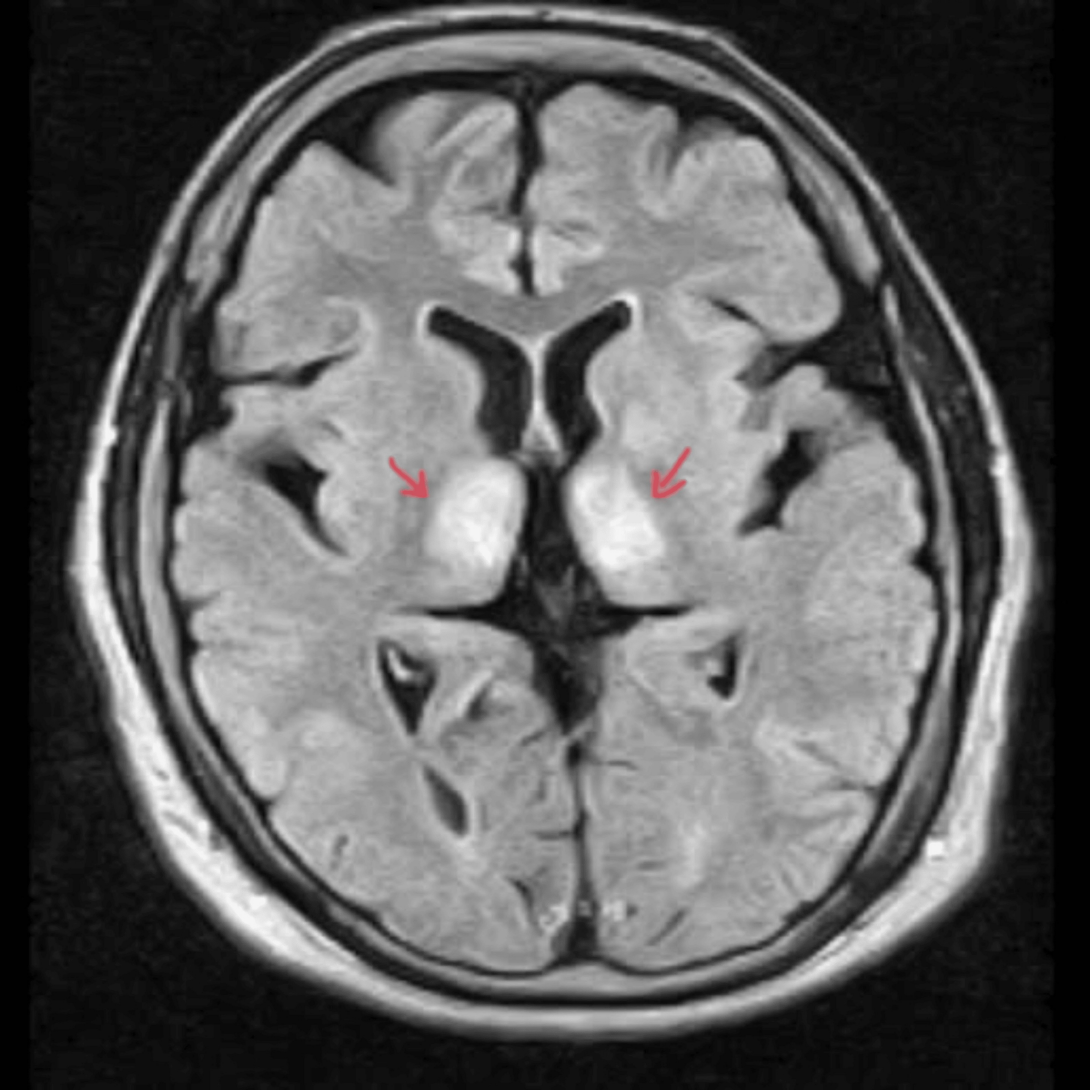
MRI of the brain showing DWI hyperintensities involving the dorsal medial and pulvinar nuclei of thalami and perimesencephalic regions DWI, diffusion-weighted image

MRI of the brain with contrast was performed to confirm the suspected diagnosis, which showed bilateral symmetrical T2-weighted (Figure 1) and diffusion-weighted image (DWI) hyperintensities involving the dorsal medial and pulvinar nuclei of thalami and perimesencephalic regions (Figure 2), showing typical findings of WE [6], thus confirming our diagnosis. Cerebrospinal fluid analysis turned out to be normal, thus excluding diagnosis of tuberculosis and viral etiology of encephalopathy. The patient was continued treatment with thiamine at a dose of 250mg thrice daily intravenously and other conservative management. The patient’s condition improved symptomatically within a short period of two weeks, and she had no further complaints on follow-up.

## Discussion

WE is recognized as a clinical emergency primarily stemming from thiamine deficiency, which can be fatal but is also treatable. Caine et al. established diagnostic criteria indicating that WE can be diagnosed in any patient exhibiting two or more symptoms from the classic triad, which include ophthalmoplegia, ataxia, and altered mental status [7]. Thiamine serves as an essential cofactor for enzymes involved in glucose metabolism, particularly in the liver, where its deficiency can lead to severe neurological impairment [8].

In pregnant women, particularly those experiencing hyperemesis gravidarum, the risk of thiamine deficiency is heightened due to both depleted reserves and increased metabolic demands [9]. Hyperemesis gravidarum can lead to significant weight loss and poor nutritional intake, potentially precipitating thiamine deficiency. However, in the case presented, the patient maintained a relatively stable condition despite hyperemesis gravidarum since the first trimester.

The deterioration of her condition coincided with the onset of a fever lasting one week, suggesting that additional factors contributed to the development of WE. It is plausible that insufficient dietary intake, stemming from poor appetite, alongside abnormal glucose metabolism due to impaired liver function, may have played a role in this case. The impaired liver function could be further attributed to co-infections with leptospirosis and scrub typhus, both of which are known to cause hepatic dysfunction and febrile illness [10]. She is also a resident of a tropical fever endemic area, where agricultural practices and proximity to water bodies are more prevalent, making her more susceptible to these infections.

Imaging studies, particularly MRI, have significantly improved the diagnostic accuracy of WE. MRI can reveal characteristic changes, such as increased signal intensity in the mesencephalic tegmentum, mammillary bodies, and medial thalamus on T2-weighted and DWI sequences [11]. This imaging modality is crucial in differentiating WE from other conditions that may present with similar clinical features. To our knowledge, this case represents a rare presentation of WE occurring in conjunction with leptospirosis and scrub typhus, highlighting the complexities involved in diagnosing and managing this condition in patients with multiple infectious and nutritional challenges.

## Conclusions

WE is a rare yet critical complication that can arise during pregnancy, particularly in patients with poor nutritional status and conditions such as hyperemesis gravidarum. Given the increased metabolic demands and potential for nutritional deficiencies in these patients, clinicians must maintain a high index of suspicion for WE. Early recognition and prompt initiation of thiamine replacement therapy are essential to improve patient outcomes and mitigate the risk of severe neurological sequelae.

The consequences of delayed diagnosis and treatment can be dire, with the potential for rapid progression to serious complications, including motor deficits, Korsakoff syndrome, coma, and even death. This highlights the importance of thorough clinical evaluation and consideration of WE in any pregnant patient presenting with symptoms indicative of neurological dysfunction, especially when accompanied by signs of malnutrition or infection.

In this context, understanding the interplay between thiamine deficiency, hepatic impairment, and concurrent infections such as leptospirosis and scrub typhus can provide valuable insights for clinicians managing complex cases. Given the rarity of WE in pregnancy, further research and awareness are warranted to ensure timely intervention and improve the prognosis of affected individuals.
